# Clinical evaluation of cardiac effects of experimental doxycycline overdosing in healthy calves

**DOI:** 10.1186/1746-6148-7-40

**Published:** 2011-07-25

**Authors:** Mounir Brihoum, Frédéric Rollin, Daniel Desmecht, Johann Detilleux, Hélène Amory

**Affiliations:** 1 Department of Companion Animals and Equids, Equine Clinic, B41, Faculty of Veterinary Medicine, University of Liège, (20, Boulevard de Colonster, Sart Tilman), Liège, (4000), Belgium; 2 Clinical Department of Production Animals, Clinic for Ruminants, B42, Faculty of Veterinary Medicine, University of Liège, (20, Boulevard de Colonster, Sart Tilman), Liège, (4000), Belgium; 3 Department of Morphology and Pathology, Systemic Pathology, B43, Faculty of Veterinary Medicine, University of Liège, (20, Boulevard de Colonster, Sart Tilman), Liège, (4000), Belgium; 4 Quantitative Genetics, B43, Faculty of Veterinary Medicine, University of Liège, (20, Boulevard de Colonster, Sart Tilman), Liège, (4000), Belgium

## Abstract

**Background:**

Cardiac morphologic and functional changes consistent with cardiomyopathy have been reported in field cases of calves with accidental doxycycline overdosing. The purpose of this study was to evaluate clinically the cardiac effects of an experimentally-induced doxycycline overdosing in healthy calves.

Twelve 2 months-old healthy Belgian Blue calves were studied. Six of them (group 1) received the normal dose (5 mg/kg, BID) and the six others (group 2) received five times the normal dose (25 mg/kg, BID) of oral doxycycline for five consecutive days (D1 to D5). Each calf was clinically examined daily. Measurement of serum AST, CK, Iso-CKs and LDH activities and an echocardiographic examination were performed before (D0) and one day after (D6) the last doxycycline administration. An ECG tracing was recorded at D0, D4, and D6.

**Results:**

In both groups, no clinical, blood, echocardiographic or electrocardiographic changes suggestive of a cardiomyopathy were observed. Only a decreased appetite was observed in the calves of the group 2 between D3 and D6.

**Conclusions:**

This trial failed to reproduce cardiac changes reported in accidental doxycycline-poisoning in calves, suggesting that high doses of doxycycline may not be the only etiologic factor of the cardiomyopathy reported in the field cases.

## Background

Doxycycline is an excellent broad-spectrum antibiotic for the treatment of respiratory diseases in calves [1]. It is obtained semi-synthetically from oxytetracycline or methacycline [2] and exerts a bacteriostatic effect by inhibiting protein synthesis [3]. This molecule presents better pharmacological properties than other tetracyclines [4], with an excellent penetration into tissues [3] and a prolonged biological half-life [5]. Moreover, the possibility of administering doxycycline to calves in milk or milk replacer makes the treatment practically easy in the field [1].

Several cases of severe, sometimes lethal, troubles were reported in 2 to 16-weeks old calves after oral intake of high doses of doxycycline [6-12]. In these calves, clinical examination revealed mainly depression, dyspnoea, cough, tongue paresia or paralysis associated to dysphagia and sialorrhea, tachycardia, arrhythmias, tachypnea and signs of myopathy [11,12]. Blood analysis revealed an increase in creatine kinase (CK), lactate dehydrogenase (LDH), aspartate aminotransferase (AST) and sorbitol dehydrogenase (SDH) activities and in creatinine and urea levels. Electrocardiographic (ECG) records and Doppler echocardiography examination revealed ventricular premature beats and a decrease in left ventricular global and systolic function, respectively. Necropsy and histopathology revealed evidence of necrosis of the myocardium, of the tongue, of the skeletal and the respiratory muscles, and acute tubular necrosis in the kidneys and fatty degeneration or congestion of the liver [6-12].

Cardiac dysfunction and necrotic cardiomyopathy lesions after treatment with doxycycline have never been described in other species including human beings, either at normal doses or at overdosing. This could suggest that cardiotoxicity of doxycycline could be specific to the bovine species, and/or that predisposing factors could play a role in the doxycycline-induced myocardial necrosis observed in calves in the field.

The purpose of this study was to evaluate clinically the cardiac effects of a normal and a high dose of doxycycline in healthy 2 months-old Belgian Blue (BB) calves.

## Methods

This study was performed according to the rules of Good Laboratory Practice and Good Clinical Practice (99/12/EEC and CPMP/ICH/135/95). The experimental protocol used in this study followed the guidelines and has been approved by the animal ethical committee of the University of Liege (Ref. 966). Only calves considered healthy on the basis of clinical history and examination were included in the trial. It was decided that calves that may show a dramatic decrease in their general status during the experimentation will be immediately euthanized. The study was conducted as blind-trial.

### Animals

Twelve BB healthy calves were studied and randomly assigned to two groups. The day before starting the experimental protocol (D0), the groups consisted of

Group 1: 6 calves (3 males and 3 females) that were 60 ± 0 days old and weighing 77 ± 5 kg.

Group 2: 6 calves (3 males and 3 females) that were 59 ± 1 days old and weighing 73 ± 7 kg.

### Housing and feeding

During the experimentation, all calves were housed in 2.2 m^2 ^rubber mats floored individual boxes. Ambient temperature and relative humidity were maintained between 10 and 15°C, and between 70 and 80%, respectively. From birth to the end of experimentation, the calves of the two groups were fed with a commercial milk replacer (Navobi^®^, Netherlands). At D0, all the calves received 640 g of this conventional milk replacer dissolved in 5 litres twice daily. During the experimentation, the amount of milk powder was increased daily of 10 g/meal. At D5, the milk powder was dissolved in 5.5 litres/meal. All calves were kept on milk only and were fed according to the same feeding schedule.

### Doxycycline administration

Calves of group 1 received a standard dosage (5 mg/kg BW) of oral doxycycline (DOXYVETO 50% PULVIS^®^; VMD) for five consecutive days (D1 to D5) BID, whilst calves of group 2 received 5-fold the standard dosage (25 mg/kg BW) of the same oral doxycycline for five consecutive days (D1 to D5) BID. Doxycycline was mixed to milk replacer and administrated to calves during feeding time. Calves were nourished individually in order to ensure that they all received the planned doxycycline dose.

### Clinical examination

All calves underwent a daily clinical examination from D0 to one day after the last doxycycline administration (D6) of the experimentation. Animals were examined 2 hours following their morning meal and before other investigations (ECG, blood samples or echocardiography) in order to avoid an effect of handling stress on their physiological parameters. The daily clinical examination of the calves was performed by an experienced veterinarian. The same operator examined all the calves during all the study and was blind to the treatment of the calves.

### Blood analysis

Blood was sampled in all calves at D0 and D6 using a venous jugular catheter. Blood was sampled in order to measure serum CK, CK isoenzymes (Iso-CKs) including muscle (Iso-CK MM), heart (Iso-CK MB), brain (Iso-CK BB) and mitochondrial (Iso-CK MT) Iso-CKs, LDH and AST activities. Measurements were performed using commercial kits (Ecoline25^®^, Ecoline15^®^, and Granutest25^® ^for AST, LDH, and CK, respectively; Diagnostica Merk). Iso-CKs were evaluated by electrophoresis.

### Electrocardiography

ECG tracings were recorded in all calves at D0, D4, and D6 from a bipolar base-apex lead (Cardiofax V, Model ECG-8240; Nihon Kohden) [13]. The electrocardiogram was only recorded when the calves were resting. The ECG tracings were analyzed manually for detection of rhythm abnormalities.

### Echocardiographic examination and measurements

An echocardiography was carried out on all calves at D0 and at D6 with a 2.5 MHz phased-array sector scanner (RT6800; GE Medical Systems) and recorded on VHS tapes for subsequent analysis. The operator performing the echocardiography was blind to the treatment of the calves. Terminology and image orientation were those recommended by the Echocardiography Committee of the Specialty of Cardiology, American College of Veterinary Internal Medicine [14] and adapted for large animals [15]. Calves were examined standing. An ECG was recorded simultaneously with the echocardiographic images.

Diastolic measurements were made at the onset of the QRS complex or at largest left ventricular dimension. Systolic measurements were made at smallest left ventricular dimension (two-dimensional [2D-] mode) or peak downward point of septal motion (time-motion [M-] mode).

The interventricular septal thicknesses (IVS) and the left ventricular internal diameter (LVID) were measured at end-diastole (d) and at end-systole (s) in an M-mode right parasternal short-axis view of the left ventricle at the level of the papillary muscles and the chordae tendinae. From these measurements, the fractional shortening (FS) of the left ventricle was calculated using the classical formula [15]. From LVID, end-diastolic (EDV) and end-systolic (ESV) volumes were calculated using the Teicholz method [16] as follows:

The ejection fraction (EF) was calculated using the classic formula [15]. The stroke index (SI) was calculated from the difference between EDV and ESV divided by the calf's BW [15]. Cardiac index (CI) was calculated from SI and HR [15].

### Statistic analysis

Results of clinical biology and echocardiography were analysed using a Statistical Analysis System (SAS) software. A mixed model for repeated data was used. The model included the effect of "day", "group" and the interaction "day" and "group". Differences were considered significant if p ≤ 0.05.

## Results

### Clinical examination

Appetite was depressed in calves of group 2 from D3 until D6 of the experimentation. The careful examination of the tongue and the evaluation of suckle response did not reveal significant findings. However, despite this appetite decrease, calves ingested the whole planned doxycycline dose. No other significant clinical modifications were observed during the experimentation.

### Blood analysis

Serum enzymology results are shown in table 1. There was no significant change in CK, Iso-CKs, AST and LDH activities between D0 and D6 regardless of the dosage of doxycycline received.

**Table 1:** Blood analysis results before (D0) and after (D6) 5 days administration of 5 mg/kg (Group 1) or 25 mg/kg (Group 2) of oral doxycycline BID in 2 months-old healthy Belgian Blue calves.

		Group 1 (n = 6)		Group 2 (n = 6)		
	**Reference** **range**	**D0** ***Mean ± SD***	**D6** ***Mean ± SD***	**D0** ***Mean ± SD***	**D6** ***Mean ± SD***	**Group 1 vs Group 2** ***p***

AST (IU/L)	70-130	77 ± 13	77 ± 16	62 ± 19	68 ± 23	NS
LDH (IU/L)	1691-2618	2464 ± 512	2578 ± 428	2031 ± 336	2169 ± 348	NS
CK (IU/L)	128-244	270 ± 257	306 ± 27	145 ± 37	191 ± 79	NS
Iso-CK MM (%)		55 ± 17	45 ± 21	39 ± 11	47 ± 16	NS
Iso-CK BB (%)		5 ± 2	4 ± 2	5 ± 2	3 ± 1	NS
Iso-CK MB (%)		3 ± 3	3 ± 2	3 ± 1	2 ± 1	NS
Iso-CK MT (%)		37 ± 14	49 ± 18	54 ± 12	49 ± 17	NS

AST: aspartate aminotransferase; LDH: lactate dehydrogenase; CK: creatine kinase; Iso-CK MM: muscle creatine kinase isoenzyme; Iso-CK BB: brain creatine kinase isoenzyme; Iso-CK MB: heart creatine kinase isoenzyme; Iso-CK MT: mitochondrial creatine kinase isoenzyme; NS: not significant.

### Electrocardiography

At D0, D4 and D6, no pathologic arrhythmias were observed in any animal.

### Echocardiography

Echocardiographic results are shown in Table 2. No significant morphological or functional echocardiographic changes were observed within or between groups.

**Table 2:** Echocardiographic parameters measured before (D0) and after (D6) administration of 5 mg/kg (Group 1) or 25 mg/kg (Group 2) of oral doxycycline BID during 5 consecutive days in 2 months-old healthy Belgian Blue calves.

	Group 1 (n = 6)		Group 2 (n = 6)		
	**D0** ***Mean ± SD***	**D6** ***Mean ± SD***	**D0** ***Mean ± SD***	**D6** ***Mean ± SD***	**Group 1 vs Group 2** ***p***

HR (beats/min)	108 ± 13	110 ± 10	83 ± 12	95 ± 27	NS
LVIDs (cm)	3.96 ± 0.42	3.65 ± 0.51	3.29 ± 0.61	3.40 ± 0.56	NS
LVIDd (cm)	5.67 ± 0.28	5.45 ± 0.46	4.91 ± 0.62	5.09 ± 0.64	NS
IVSs (cm)	1.86 ± 0.11	1.88 ± 0.10	1.84 ± 0.17	1.83 ± 0.17	NS
IVSd (cm)	1.11 ± 0.03	1.11 ± 0.10	1.12 ± 0.18	1.16 ± 0.14	NS
FS (%)	30.19 ± 6.74	33.22 ± 4.43	33.35 ± 6.25	33.40 ± 5.48	NS
% IVS	67.05 ± 6.74	69.91 ± 8.59	65.63 ± 20.28	58.33 ± 17.06	NS
EDV (cm^3^)	158.40 ± 17.90	145.50 ± 19.99	115.51 ± 9.24	125.65 ± 22.68	NS
ESV (cm^3^)	69.13 ± 17.20	57.72 ± 14.50	45.73 ± 12.27	49.08 ± 16.16	NS
SI (ml kg^-1^beat^-1^)	1.17 ± 0.27	1.01 ± 0.20	0.95 ± 0.14	1.04 ± 0.16	NS
CI (ml kg^-1 ^min^-1^)	0.13 ± 0.04	0.11 ± 0.04	0.08 ± 0.03	0.10 ± 0.03	NS
EF	0.56 ± 0.09	0.61 ± 0.05	0.61 ± 0.06	0.61 ± 0.06	NS

HR: heart rate; LVIDs and LVIDd: left ventricular internal diameter in systole and diastole; IVSs and IVSd: interventricular septum thickness in systole and diastole; FS: fractional shortening of the left ventricle; %IVS: fractional thickening of the interventricular septum; EDV: end diastolic volume; ESV: end systolic volume; SV: stroke volume; SI: stroke index; CI: cardiac index; EF: ejection fraction.

## Discussion

Several cases of doxycycline intoxication have been reported in calves aged between 2 and 16 weeks, originating from different breeds (including BB calves), and that received 3 to 10 times the recommended dose of doxycycline [6-12]. In these cases, cardiac toxicity was suggested by clinical signs (tachycardia, arrhythmias, sudden death) and post-mortem examination (myocardial necrosis). The purpose of the present study was to experimentally reproduce the same troubles in calves with age and breed comparable to those described in previous papers. The major limitation of the present study is the fact that no post-mortem examination was performed. However, cardiac function of the studied calves was investigated through clinical examination, ECG, echocardiography and serum enzymology.

Previous studies on accidental doxycycline intoxication in calves reported untoward clinical signs, leading to sudden death in some cases, 1 to 5 days after oral doxycycline overdosing [6-9,11,12]. In this experimental doxycycline overdosing, only a decreased appetite was clinically observed in doxycycline-overdosed calves. A similar appetite decrease has also been previously reported in field occurring cases of doxycycline-overdosing [8,9]. Clinical evolution of the studied doxycycline-overdosed calves was thus clinically completely distinct from the clinical pattern reported in accidental cases.

No clinical signs suggestive of cardiomyopathy (such as those reported by previous authors in field occurring cases of doxycycline-overdosing) were detected after 5 days of doxycycline administration at 5 times the recommended dose in the present study. Cardiac arrhythmias are among the most common signs observed with cardiomyopathy in cattle [17,18], cats [19], dogs [20] and in human beings [21]. Cardiac arrhythmias were also reported in accidentally field-occurring doxycycline overdosed calves [9,11,12]. On the contrary, in the present experimental doxycycline overdosing, no cardiac arrhythmias were detected during the daily clinical examination or on ECG examination performed after 3 and 5 days of doxycycline administration. However, it would have been better to perform a holter examination of the investigated calves to exclude with greater certainty the occurrence of cardiac arrhythmias.

Spectacular elevation of serum muscular enzymes activity was reported in previous cases of doxycycline intoxication in calves [9,12], suggesting skeletal muscles damage possibly associated with myocardial damage [22,23]. In the present study, clinical biology revealed absolutely no modifications in serum CK, Iso-CKs, LDH, Iso-LDHs and AST activities between D0 and D6 in calves that received either normal or high dose of doxycycline. These results strongly suggest that no muscle damage (including skeletal muscles and myocardium) occurred between D0 and D6. Cardiac isoenzymes of LDH and CK were amongst the first utilized cardiac markers in human medicine [24]. However, those cardiac biomarkers have been shown to have a lack of specificity and a low sensitivity, so they are almost no longer used in human patients. In veterinary medicine, isoenzymes 1 and 2 of lactate dehydrogenase and the MB fraction of creatine kinase are still used because until recently, there were no alternative cardiac biomarkers validated in animals. In the recent years, the measurement of serum cardiac troponin I or T has emerged as the biochemical "gold standard" for diagnosis of patients suffering from myocardial injury [25-27]. More recently, the measurement of this marker has been validated in several animal species, and it has been showed to be increased in animals, including cattle [28], suffering from various cardiac diseases. The use of cardiac troponin I was validated in cattle [29], but bovine cardiac troponin T was not accurately quantified with a common human clinical immunoassay [30]. It would thus have been interesting to use cardiac troponin I in the present study.

In this study, no cardiac morphological or functional modifications were observed by echocardiography between D0 and D6 in the 2 groups.

Left ventricular FS, EF and cardiac output (CO) are common measurements of left ventricular systolic and global function [15,31,32] and are expected to undergo changes when myocardial performance is impaired [15]. In the present study, parameters of systolic function and cardiac global performance (SI and CI) were preserved even in overdosed calves. These results suggest that overdosing doxycycline in healthy BB calves did not affect their cardiac function.

Therefore, the clinical, biochemical, electrocardiographical and echocardiographical results of this study suggest that an experimentally-induced doxycycline overdosing at 5 times the normal dose for 5 consecutive days in healthy calves did not reproduce the cardiomyopathy that has been reported in several calves receiving high doses of doxycycline in the field. This means that in the previous outbreaks of doxycycline-poisoning in calves, overdosing doxycycline was not the only cause of the reported myocardial necrosis. Some other exacerbating factors may play a role in the development of this process.

Even if reported in a small number of calves, clinical biology performed in doxycycline accidentally-overdosed calves revealed low levels of blood vitamin E (one calf out of two sampled) and selenium (two calves out of two sampled) content [9]. On the other hand, post mortem findings in doxycycline poisoned calves are very similar to observations made on calves suffering from vitamin E/selenium deficiency-associated myopathy and/or cardiomyopathy. Vitamin E/selenium deficiency could thus be a potential exacerbating factor and a potential link between doxycycline-overdosing and vitamin E/selenium metabolism may exist. Interaction between doxycycline and other drugs may also be possible. Finally, a delayed cardiac toxicity could also be suggested. In some previous reports, signs of doxycycline toxicity were observed after five days of doxycycline administration [6]. Even if reported only in one paper, this observation should not be ignored as well as the fact that there can be individual variation among calves tolerance to this drug.

## Conclusions

Overdosing doxycycline at 5 times the recommended dose for 5 consecutive days in healthy 2 months-old BB calves did not result in clinical, electrocardiographic or echocardiographic signs of cardiomyopathy. Therefore, the myocardial lesions found in calves accidentally overdosed with doxycycline may be attributable to other yet unknown factors.

## Authors' contributions

MB, FR, DD and HA performed the experiments and analyzed the data. JD performed statistical analysis of data. All authors have read and approved the final manuscript.

## References

[B1] Van Gool F Santoul C Raynaud JP Caracteristiques pharmacocinetiques et bilan des essais cliniques pour le traitement ou la metaphylaxie des bronchopneumonies infectieuses des veux de boucherie par le Ronaxan^®^ Proceedings of the 14th World Congress on Diseases of Cattle, Dublin 1986 1 627 631

[B2] Riond JL Riviere JE Pharmacology and toxicology of doxycycline Vet Hum Toxicol 1988 30 431 443 3055652

[B3] Shaw DH Rubin SI Pharmacologic activity of doxycycline J Am Vet Med Assoc 1986 189 808 810 3771346

[B4] Meijer LA Ceyssens KGF Greve BIJACd Bruijn Wd Pharmacokinetics and bioavailability of doxycycline hyclate after oral administration in calves Vet Q 1993 15 1 5 8498009 10.1080/01652176.1993.9694358

[B5] Papich MG Rivière JE Riviere JE, Papich MG, Adams HR Tetracycline antibiotics Veterinary pharmacology and therapeutics 2009 9 Ames, Iowa: Wiley-Blackwell 895 913

[B6] Zeeuwen AAPA Van Exsel ACA Jaartsveld FHJ Wentink GH Doxycycline-vergiftiging bij witvleeskalveren Tijdschr Diergeneeskd 1993 118 803 8278981

[B7] Mc Ewen B Lusis P Archambault M Van Dreumel T Doxycycline cardiotoxicity in veal calves AHL Newsletter 2002 6 5

[B8] Yeruham I Perl S Sharony D Vishinisky Y Doxycycline toxicity in calves in two feedlots J Vet Med Ser B 2002 49 406 408 10.1046/j.1439-0450.2002.00597.x 12449251

[B9] Brihoum M Amory H Desmecht D Rollin F Doxycycline poisoning in calves: 18 cases in Belgium Proceedings of the 23rd World Buiatrics Congress: 2004 2004 Québec, Canada 102

[B10] Brihoum M Cassart D Amory H Rollin F Desmecht D Post mortem findings in 22 doxycycline overdosed calves referred for sudden death Proceedings of the 23rd World Buiatrics Congress 2004 Québec, Canada 102

[B11] Chiers K Weyens P Deprez P Van Heerden M Meulemans G Baert K Croubels S De Backer P Ducatelle R Lingual and pharyngeal paralysis due to acute doxycycline intoxication in veal calves Vet Rec 2004 155 25 26 10.1136/vr.155.1.25 15264487

[B12] Brihoum M Amory H Desmecht D Cassart D Deleuze S Rollin F Descriptive Study of 32 Cases of Doxycycline-Overdosed Calves J Vet Intern Med 2010 24 1203 1210 10.1111/j.1939-1676.2010.0560.x 20673319

[B13] Amory H Rollin F Genicot B Beduin JM Lekeux P Comparative study of the body surface ECG in double-muscled ans conventional calves Can J Vet Res 1993 57 PMC1263614 8358673

[B14] Thomas WP Gaber CE Jacobs GJ Kaplan PM Lombard CW Moise NS Moses BL Recommendations for standards in transthoracic two-dimensional echocardiography in the dog and cat J Vet Intern Med 1993 7 247 252 10.1111/j.1939-1676.1993.tb01015.x 8246215

[B15] Boon JA Boon JA The Echocardiographic Examination Manual of veterinary echocardiography 1998 Baltimore: Williams and Wilkins 35 236

[B16] Teicholz LE Kreulen T Herman MV Problems in echocardiographic volume determinations: echocardiographic-angiographic correlations in the presence or absence of asynergy Am J Cardiol 1976 37 7 11 10.1016/0002-9149(76)90491-4 1244736

[B17] Van Vleet JF Amstutz HE Weirich WE Rebar AH Ferrans VJ Clinical, clinicopathologic, and pathologic alterations in acute monensin toxicosis in cattle Am J Vet Res 1983 44 2133 2144 6650960

[B18] Shlosberg A Harmelin A Perl S Pano G Davidson M Orgad U Kali U Bor A Ham Mv Hoida G Cardiomyopathy in cattle induced by residues of the coccidiostat maduramicin in poultry litter given as a feedstuff Vet Res Commun 1992 16 45 58 10.1007/BF01839204 1598754

[B19] Fox PR Fox PR, Sisson D, Moise NS Feline cardiomyopathy Textbook of canine and feline cardiology: principles and clinical practice 1999 2 Philadelphia: Saunders 621 678

[B20] Sisson D O'Grady MR Calvert CA Fox PR, Sisson D, Moise NS Myocardial diseases of dogs Textbook of canine and feline cardiology: principles and clinical practice 1999 2 Philadelphia: Saunders 581 619

[B21] Richardson P Mc Kenna W Bristow M Maisch B Mautner B O'Connel J Olsen E Thiene G Goodwin J Gyarfas I Report of the 1995 World Health Organization/International Society and Federation of Cardiology Task Force on the Definition and Classification of cardiomyopathies Circulation 1996 93 841 842 8598070 10.1161/01.cir.93.5.841

[B22] Anderson PH Bradley R Berrett S Patterson DSP The sequence of myodegeneration in nutritional myopathy of the older calf Br Vet J 1977 133 160 165 608055 10.1016/s0007-1935(17)34137-4

[B23] Gozalo AS Chavera A Montoya EJ Takano J Weller RE Relationship of creatine kinase, aspartate aminotransferase, lactate dehydrogenase, and proteinuria to cardiomyopathy in the owl monkey (Aotus vociferans) J Med Primatol 2008 37 29 38 18269526 10.1111/j.1600-0684.2007.00258.x

[B24] Jaffe A Landt Y Parvin C Abendschein D Geltman E Ladenson J Comparative sensitivity of cardiac troponin I and lactate dehydrogenase isoenzymes for diagnosing acute myocardial infarction Clin Chem 1996 42 1770 1776 8906075

[B25] Rosenbaum LS Januzzi JL Moving troponin testing into the 21st century: will greater sensitivity be met with greater sensibility? Cardiovascular & Hematological Disorders-Drug Targets 2008 8 118 126 18537598 10.2174/187152908784533711

[B26] White HD Chew DP Acute myocardial infarction The Lancet 2008 372 570 584 10.1016/S0140-6736(08)61237-4 18707987

[B27] Wu AHB Jaffe AS The clinical need for high-sensitivity cardiac troponin assays for acute coronary syndromes and the role for serial testing Am Heart J 2008 155 208 214 10.1016/j.ahj.2007.10.016 18215588

[B28] Mellanby RJ Henry JP Cash R Ricketts SW Bexiga R Truyers I Mellor DJ Serum cardiac troponin I in cattle with cardiac and noncardiac disorders J Vet Intern Med 2009 23 926 930 10.1111/j.1939-1676.2009.0330.x 19496907

[B29] Varga A Schober KE Walker WL Lakritz J Rings DM Validation of a commercially available immunoassay for the measurement of bovine cardiac troponin I J Vet Intern Med 2009 23 359 365 10.1111/j.1939-1676.2009.0256.x 19192157

[B30] Willis MS Snyder JA Poppenga RH Grenache DG Bovine cardiac troponin T is not accurately quantified with a common human clinical immunoassay J Vet Diagn Invest 2007 19 106 109 10.1177/104063870701900119 17459843

[B31] Dyson DH Allen DG McDonell WN Comparison of three methods for cardiac output determination in cats Am J Vet Res 1985 46 2546 2552 4083591

[B32] Knowlen GG Olivier NB Kittleson MD Cardiac contractility. A review J Vet Intern Med 1987 1 188 198 10.1111/j.1939-1676.1987.tb02014.x 3333413

